# Polyaromatic Hydrocarbon Inclusion Complexes with 2-Hydroxylpropyl-β/γ-Cyclodextrin: Molecular Dynamic Simulation and Spectroscopic Studies

**DOI:** 10.3390/molecules29112535

**Published:** 2024-05-28

**Authors:** Norah S. Alsadun, Amira A. Alfadil, Abdalla A. Elbashir, FakhrEldin O. Suliman, Mei Musa Ali Omar, Amel Y. Ahmed

**Affiliations:** 1 Department of Chemistry, College of Science, King Faisal University, Al-Ahsa 31982, Saudi Arabia; aebrahim@kfu.edu.sa; 2 Department of Chemistry, College of Science, Sultan Qaboos University, P.O. Box 36, Al-Khoud 123, Oman; 3 Department of Chemistry, Faculty of Science, University of Khartoum, Khartoum 11114, Sudan; 4 Department of Scientific Laboratories, College of Science, Sudan University of Science and Technology, Khartoum 11115, Sudan; 5 Central Laboratory, Department of Chemistry, Ministry of Higher Education & Scientific Research, Khartoum 7099, Sudan; maichemistry@hotmail.com

**Keywords:** cyclodextrin, inclusion complexes, molecular dynamics, polyaromatic hydrocarbons, solid complex

## Abstract

In aqueous and solid media, 2-HP-β/γ-CD inclusion complexes with poly aromatic hydrocarbon (PAH) Phenanthrene (PHN), Anthracene (ANT), Benz(a)pyrene (BaP), and Fluoranthene (FLT) were investigated for the first time. The inclusion complexes were characterized and investigated using fluorescence and ^1^HNMR spectroscopy. The most prevalent complexes consisting of both guests and hosts were those with a 1:1 guest-to-host ratio. The stability constants for the complexes of PHN with 2-HP-β-CD and 2-HP-γ-CD were 85 ± 12 M^−1^ and 49 ± 29 M^−1^, respectively. Moreover, the stability constants were found to be 502 ± 46 M^−1^ and 289 ± 44 M^−1^ for the complexes of ANT with both hosts. The stability constants for the complexes of BaP with 2-HP-β-CD and 2-HP-γ-CD were (1.5 ± 0.02) × 10^3^ M^−1^ and (9.41 ± 0.03) × 10^3^ M^−1^, respectively. The stability constant for the complexes of FLT with 2-HP-β-CD was (1.06 ± 0.06) × 10^3^ M^−1^. However, FLT was observed to form a weak complex with 2-HP-γ-CD. Molecular dynamic (MD) simulations were used to investigate the mechanism and mode of inclusion processes, and to monitor the atomic-level stability of these complexes. The analysis of MD trajectories demonstrated that all guests formed stable inclusion complexes with both hosts throughout the duration of the simulation time, confirming the experimental findings. However, the flexible Hydroxypropyl arms prevented the PAHs from being encapsulated within the cavity; however, a stable exclusion complex was observed. The main forces that influenced the complexation included van der Waals interactions, hydrophobic forces, and C–H⋯π interaction, which contribute to the stability of these complexes.

## 1. Introduction

**Figure 1:**
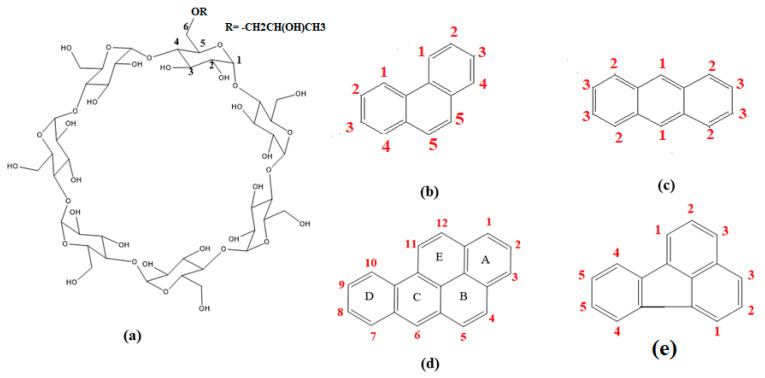
The structure and numbering of the atoms of (**a**) β-CD, (**b**) PHN, (**c**) ANT, (**d**) BaP and (**e**) FLT.

Cyclodextrins (CDs) are cyclic oligosaccharides consisting of six (α-CD), seven (β-CD), or eight (γ-CD) D-(+)-glucopyranose units, which are connected at 1 and 4 carbon atoms by glycosidic bonds [1]. On the other hand, while those with nine to twelve units have been reported, their complexing ability appears to be poor [2]. CDs have a cone shape due to a lack of free rotation about the bonds connecting the glucopyranose units. An interesting structural feature is that hydroxyl groups decorate the two entrances of this cone (Figure 1). The secondary hydroxyl groups are positioned along the broader edge, whilst the primary hydroxyl groups are situated along the narrower side of the torus. In some instances, these primary hydroxyl groups can block the narrow rim partially due to their ability to freely rotate, leading to the reduced complexation efficiency of this side [3,4]. CDs are water-soluble compounds; this positions them at the forefront of a diverse array of applications. β-CD is less soluble than the other types owing to its capacity to form intramolecular hydrogen bonds, which counteract its hydration due to the rigid structure. The water solubility of β-CD is 18.5 mg mL^−1^, whereas it is 232 mg mL^−1^ for γ-CD at 25 °C [5]. Various hydrophilic, hydrophobic, and ionic CD derivatives have been successfully synthesized and commercialized to promote the aqueous solubility of CDs. The alkylation and hydroxyl alkylation of the hydroxyl groups were achieved to produce 2-hydroxypropyl-β-CD with desirable properties, like a low toxicity profile, high water solubility, and amorphous nature, which extends its applications in various fields. The alkaline condensation of the parent CDs with propylene oxide produces HP-β/γ-CD in pure form, with different degrees and positions of substituents [6,7]. The unique structure of CDs renders them powerful complexing and solubilizing agents. They can form inclusion complexes of the host–guest type with a wide variety of guest molecules by encapsulating a whole molecule or part of it into the hydrophobic cavity through non-covalent interaction. This may result in many changes in the physicochemical properties of the molecules. The ability of CDs to form inclusion complexes leads to their widespread application in pharmaceutical chemistry, analytical chemistry, food technology, chemical synthesis, and catalysis [8,9]. Several forces act together to form a stable host–guest complex, such as hydrogen bonding interactions, hydrophobic interactions, and van der Waals forces. The extent to which each is involved depends on the nature of the guests [10]. The driving forces for inclusion complexation are both enthalpy and entropy dependent. When the guest molecule enters the cyclodextrin cavity, it loses its hydration sphere, and the water molecules are expelled from the cavity, resulting in a rise in entropy and enthalpy [11]. Complexes of various stoichiometry (guest–host) have been reported, such as 1:1, 1:2, 2:1 and 2:2 [12,13].

CDs are used as stabilizers, delivery systems, drug carriers, solubilizing agents, and the building blocks for the construction of supramolecular complexes [14]. Moreover, CDs can serve as excipients for food, pharmaceuticals, cosmetics, and other applications [3,15,16]. 

CDs and their derivatives have found extensive application in numerous spectrofluorimetric techniques. They augment the fluorescence intensity of many guest molecules when encapsulated partially or totally by shielding them from quenching reactions and non-radiative decay processes. Aqueous β/γ-CD was reported as a molecular organizing media for analyses based on the luminescence of the compounds [17]. Surprisingly, although extensive research has been carried out on CDs, no single study has examined the association between unsubstituted PAHs and CDs and attempted to investigate their association and stability. 

Polynuclear aromatic hydrocarbons are a group of chemical compounds containing two or more fused aromatic rings bonded in linear, cluster, or angular arrangements. They are made of carbon and hydrogen atoms only [18]. The existence of PAHs in nature originated either from anthropogenic or natural sources [19]. Anthropogenic sources can be derived from the incomplete combustion or pyrolysis of organic matter by a series of complex chemical reactions [20]. Meanwhile, the natural sources are from plant chlorophyll, bacteria, and fungus [19]. PAHs have versatile properties and are strongly dependent on their molecular structures, which are defined by their structural configuration and molecular size [21]. PAHs exhibit typical electroluminescent and photoluminescent characteristics, as well as exceptional electrochemical capabilities. They constitute an active component in organic light-emitting diodes (OLEDs), as well as photovoltaics [22]. The lipophilic nature of PAHs facilitates their permeation across lipid membranes and subsequent accumulation within aquatic organisms. This accumulation can result in the bioaccumulation of PAHs in higher trophic levels, including top predators such as humans. Moreover, PAHs have the potential to disrupt normal DNA functioning [23]. Undoubtedly, exposure to PAHs leads to a range of adverse health effects, including the development of different types of malignancies, the disruption of endocrine systems, and the suppression of immunological function [24].

Anthracene (ANT) is composed of three benzene rings fused linearly. This molecular structure results in an extended aromatic system with conjugated π-electrons, which imparts intriguing photochemical and photophysical characteristics to anthracene. Additionally, anthracene exhibits the potential to form gels [25]. FLT is one of the most abundant PAHs in coal derivatives, such as creosote, and is found at many contaminated sites [26]. Considering the deleterious effects of PAH on human health and the environment, we have selected these four PAHs as the target of this study because they are some of the 16 priority PAHs indicated by the United States Environmental Protection Agency (UN-EPA) [27].

Recently, a substantial body of literature about the utilization of cyclodextrin as a host molecule has emerged. It is worth noting that currently, there is no existing study specifically examining the complexation of CDs with PHN, ANT, BaP, and FLT. Given that PAHs are a growing category of contaminants, there is a need to establish straightforward, eco-friendly, and precise analytical techniques for detecting minute amounts of these substances in environmental and food samples. This study provides insights into the impact of a macrocyclic molecule on the spectroscopic characteristics of these guests. It is anticipated that the interactions between the guests and the hosts will enhance the electronic characteristics of the guests, specifically their fluorescence intensities. This would enable the establishment of straightforward and precise analytical methods for quantifying them in intricate matrices, such as food samples.

## 2. Results and Discussion

### 2.1. Fluorescence Measurements

In this study, the fluorescence spectrophotometric technique was employed to evaluate the complexation of the four guests with the two hosts. In addition, we aimed to investigate the impact of this complexation on the fluorescence characteristics of these guest molecules. This could potentially lead to the development of a highly sensitive and specific analytical technique for their detection in food and environmental samples. The guests have a substantial conjugated architecture and exhibit robust intramolecular charge transfer (ICT), resulting in their possession of a significant fluorescence emission [21].

**Figure 2:**
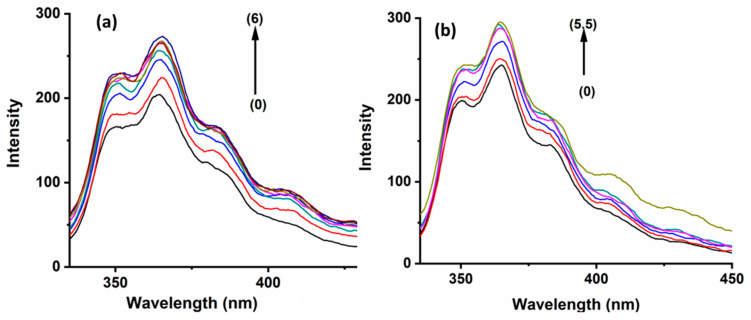
Fluorescence spectra of (0) PHN, 1.0 × 10^−6^ M with increasing concentrations of (**a**) HP-β-CD 1.0 × 10^−3^ M → 6.0 × 10^−3^ M, and increasing concentrations of (**b**) HP-γ-CD 1.0 × 10^−3^ M → 5.5 × 10^−3^ M, with an increase of increment of 0.001.

**Figure 3:**
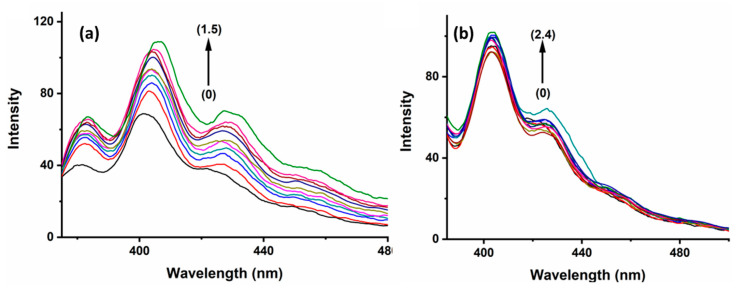
Fluorescence spectra of (0) ANT, 5.0 × 10^−7^ M with increasing concentrations of (**a**) HP-β-CD 3.0 × 10^−3^ M → 1.50 × 10^−2^ M and increasing concentrations of (**b**) HP-γ-CD 1.0 × 10^−3^ M → 2.4 × 10^−3^ M, with an increase in increment of 0.0005.

The structured emission spectra of PHN are in the wavelength range of 300 to 450 nm when excited at 292 nm, as shown in Figure 2. The ANT exhibits two inherent peaks at wavelengths of 400 and 420 nm (Figure 3). The bands mentioned above are attributed to the locally excited state characterized by a π-π* transition.

Figure 2 and Figure 3 show the fluorescence spectra of PHN and ANT in the presence of 2-HP-β/γ-CD. Both systems exhibit the same trend of an increase in emissions. The emission enhancement observed reveals that the complex formation process reduces the amount of energy wasted by the internal conversion. Moreover, the cavities provide optimal protection against the quenching effects that surround the molecules in the bulk solution [28]. The most interesting finding was that ANT has larger association constant, K, values but small fluorescence enhancement upon complexation with CDs. The literature has established that the enhancement of fluorescence quantum yield (Φ) values by the cyclodextrins inclusion complex does not correlate with the stability (K value) of the complex. Sueishi et al. clarified that the local polarity of the excited state of the guest plays a crucial role in the difference in the emission intensity and the stability of the inclusion complex [29]. However, it seems possible that the hydrophobic cavity supplies a comfortable microenvironment for the guests, increasing their fluorescence quantum yield [11].

The affinity between a host and guest in a solution can be evaluated by determining the binding constant (K). The thermodynamic stability of the host–guest combination at a specific temperature in a particular solvent can be quantified using fluorescence measurements [30]. The Bensi–Hilderbrand equation (Equation (1)) can be utilized to determine the association constant and stoichiometry of the interaction between the four guests and the two hosts.

(1) $$\frac{1}{F-F_{o}}=\frac{1}{F_{\infty }-F_{o}}+\frac{1}{\left(F_{\infty }-F_{o}\right)K{\left[host\right]}_{o}^{n}}$$

The fluorescence intensity, F, represents the measured fluorescence intensity for each tested [host]_o_. In this context, F_∞_ and F_o_ are the upper limit of the fluorescence intensity shown by the complex under investigation and the fluorescence intensity observed in the absence of the host, respectively. Additionally, K represents the association or binding constant associated with the formation of the complex.

The experimental findings demonstrate a positive correlation between the increase in the guest molecules’ fluorescence intensity and the host molecules’ concentration, indicating a suitable fit for a 1:1 complex formation. To justify our assumption, the double reciprocal plot of the host concentration vs. the reciprocal of the difference between the final fluorescence intensity (F∞) and the initial fluorescence intensity (Fo) was constructed. Table 1 summarize the results.

The binding constants for PHN and ANT with 2-HP-β-CD are larger than with 2-HP-γ-CD. The rigidity of the local environment has a crucial effect on the fluorescence emission intensity [31]. 2-HP-β-CD is more rigid than 2-HP-γ-CD. Hence, the guest molecules will experience greater restraints inside the 2-HP-β-CD cavity. Consequently, this maximizes the hydrophobic interactions with the ensuing thermodynamic stability to give a more stable complex. It could be argued that 2-HP-β-CD provides a better cavity for the PHN and ANT solution during fluorescence sensing. A comparison of the K values of the probes reveals that ANT-CD complexes are more stable than PHN-CD, even though PHN is more stable than ANT [32]. This indicates that the size, shape, and total energy of the guest play an important role in the stability of the resulting complexes. This indicates that the host–guest complexation process is stereo structurally controlled. 

**Figure 4:**
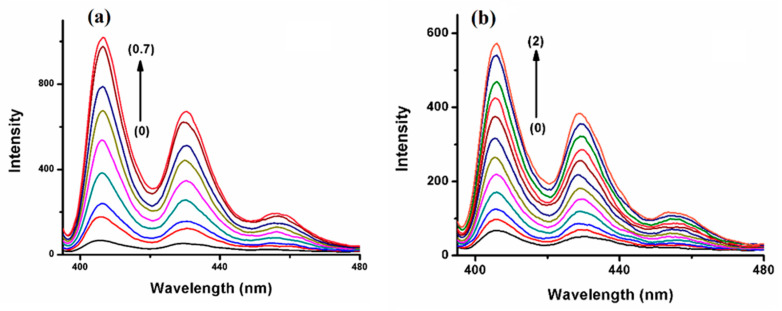
Fluorescence spectra of (0) BaP, 5.0 × 10^−7^ M with increasing concentrations of (**a**) HP-β-CD 5 × 10^−5^ M → 0.7 × 10^−3^ M. (**b**) HP-γ-CD 1.0 × 10^−4^ M → 2.0 × 10^−3^ M, with an increment of 0.0002.

BaP, when excited at 385 nm in aqueous media, exhibits two highly pronounced vibronic structure emission bands at 405 nm and 420 nm, as well as a larger and less strong band at 540 nm (Figure 4), which represents the excimer species [33]. In contrast, the FLT fluorescence spectrum shows a wavelength of excitation maxima at 350 nm, accompanied by a broad emission peak spanning from 400 to 500 nm and centered at 455 nm (Figure S1). The fluorescence emissions of BaP and FLT increase upon the gradual addition of 2-HP-β/γ-CD, which confirms that a host–guest interaction forms the inclusion complex (Figure 4 and Figure S1). It is worth mentioning here that several processes act simultaneously to contribute to the formation of stable host–guest complexes. The extent to which each of them is involved depends on the guest’s nature [34]. The driving forces that are responsible for complex formation include contributions from van der Waals interactions, electrostatic interactions, hydrophobic interactions, the release of conformational strain, hydrogen bonding, and charge transfer [35].

An entering guest feels a relatively hydrophobic surface since ether oxygen lines the cavity’s interior. Undoubtedly, the hydrophobic effect provided by the microenvironment puts the guest in a lower-energy environment. Furthermore, it shields the excited singlet species from the quenching and non-radiative decay processes present in the aqueous bulk solution, and consequently, the fluorescence emission will increase [8,36]. 

Water release from the cavity does not drive cyclodextrin complex formation; the most acceptable reason for this is that the water molecules inside the cavity have more conformational freedom, higher energy, and fewer hydrogen bonds. The release of these water molecules results in a negative enthalpy change, but the overall free energy change is not necessarily negative [37,38]. Along the same lines, Esmeralda and Maria reported that in accordance with enthalpy–entropy compensation, releasing structural strain and excluding cavity-bound high-energy water molecules do not add energy to the complex; rather, these modifications occur after complexation to enhance the host–guest interaction and establish stronger hydrophobic and van der Waals interactions [39]. FLT and BaP are nonpolar hydrophobic molecules; hence, van der Waals and hydrophobic interactions are the main driving forces of the complexation here. The binding constant K values for FLT and BaP with the 2-HP-β-CD are much larger than with 2-HP-γ-CD, which has a larger cavity (Table 1). This is because of the stronger and closer interaction between the guests’ molecules and the 2-HP-β-CD hydrophobic cavity walls. The wider cavity size of 2-HP-γ-CD (7.5 Å) produces weak attraction.

### 2.2. NMR Spectroscopy

**Figure 5:**
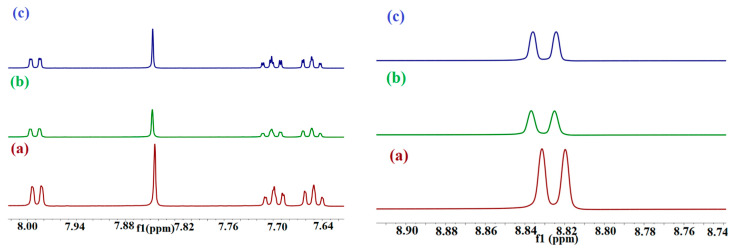
Parts of ^1^H NMR spectra of (a) pure PHN compared to (b) PHN-HP-β-CD and (c) PHN-HP-γ-CD in DMSO. The molar ratio of HP-β/γ-CD/guest is 1:1, with the **left side** from 7.64–8 ppm, and the **right side** from 8.74–8.90 ppm.

The polyaromatic PHN displays five peaks corresponding to aromatic protons, as determined by the structure depicted in Figure 1b. These peaks are observed at chemical shifts ranging from 8.825 ppm to 7.650 ppm. Notably, these chemical shifts change when PHN interacts with the hosts in DMSO, as illustrated in Figure 5.

**Figure 6:**
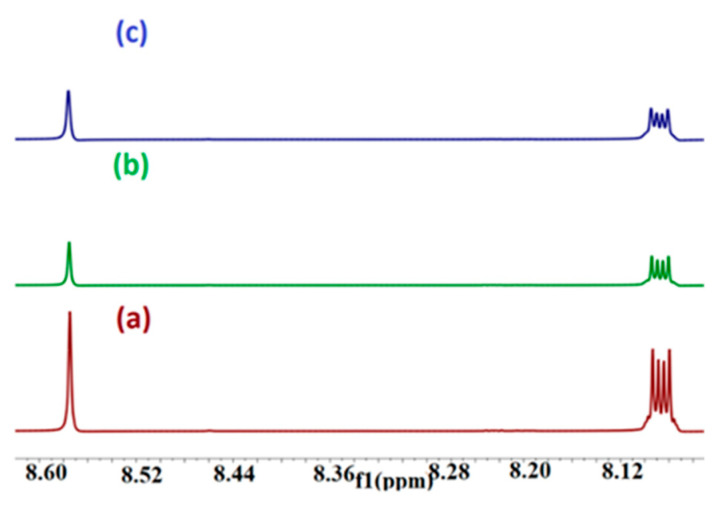
Parts of ^1^H NMR spectra of (a) pure ANT compared to (b) ANT-HP-β-CD and (c) ANT-HP-γ-CD in DMSO. The molar ratio of HP-β/γ-CD/guest is 1:1.

**Figure 7:**
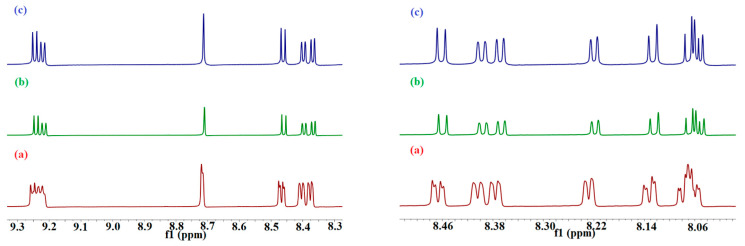
A partial ^1^H-NMR spectra for the (b) BaP–HP-β-CD and (c) BaP–HP-γ-CD complex compared to (a) free BaP in DMSO. The molar ratio of HP-β/γ-CD/guest is 1:1, with the **left side** from 8.3–9.3 ppm, and the **right side** from 8.06–8.46 ppm.

2-HP-β/γ-CD is composed of a combination of many closely related derivatives. These derivatives exhibit diverse levels of substitution and exist in different isomeric forms. As a result, the NMR peaks produced by this compound are characterized by a significant degree of broadness [40]. PHN and ANT protons have been shifted downfield upon adding 2-HP-β/γ-CD (Figure 5 and Figure 6), indicating the deshielding effect caused by oxygen atoms located in the cavity rim, which denotes the occurrence of partial guests’ insertion. The truncated shape of the CD cavity and the existence of large substituents placed on the glucose units of the CDs impart the ability to form stable inclusion complexes with the guests [41]. Small downfield shifts in protons are attributed to weak host–guest interactions [42]. In the ^1^H NMR spectrum of pure BaP, twelve distinct aromatic proton peaks range from 9.253 ppm to 7.861 ppm (Figure 7). These peaks are significantly deshielded by the massive anisotropic field generated by the electron cloud in the rings’ structure [43]. The addition of 2-HP-β/γ-CD to the BaP solution changes the surrounding electron or magnetic environment, which induces up-field spectral shifts due to the host shielding effect, Table 2.

Tables S1 and S2 show the detailed variation in the PHN and ANT protons’ chemical shifts before and after complexation with 2-HP-β/γ-CD.

The ^1^H NMR spectra of pure FLT displayed five distinct aromatic peaks, with chemical shifts ranging from 8.143 ppm to 7.438 ppm, which agrees with the reported values [44]. These values were changed following the addition of the two hosts, providing evidence to support complexation, and were shown to shift downfield upon introducing the two hosts (Figure S2). This finding is indicative of the complex formation. The observed changes towards lower magnetic fields in the protons belonging to the guest molecules can be ascribed to the alteration in the anisotropic effect exerted by their surrounding environment [40]. Following complexation, the guest molecule’s gyration radius decreases, causing the guest protons to be positioned closer to the deshielding cone of the benzene ring. Consequently, the protons experience a greater influence from the benzene ring compared to the hydrophobic shielding effect provided by the cavity. Furthermore, it is worth considering which specific side of the molecule is inserted into the cavity of the host. The answers to these queries can be obtained by referring to Table 3, which provides a summary of the value and sign of Δδ. The host molecule determines the sign of the proton shifts in the guest molecules. For example, in the FLT–CD complexes, the Δδ values are greater than zero. This suggests that they are subject to the same influences. As indicated in the existing body of research, the observed discrepancies in hydrogen chemical shifts mostly arise from disparities in the contributions of C-C (π) and C-C (σ) interactions [45].

### 2.3. Molecular Dynamic Simulations

**Figure 8:**
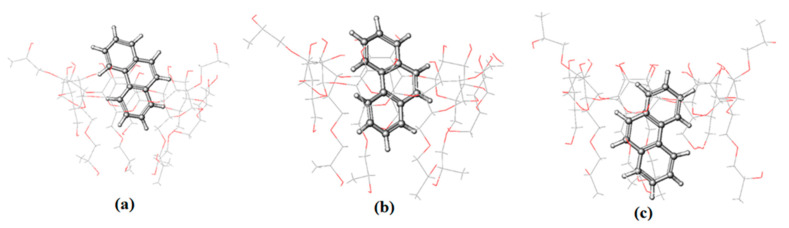
The representative snapshots of the PHN-HP-β-CD obtained during simulation at (**a**) 0 ns, (**b**) 10 ns and (**c**) 29.8 ns.

To obtain more information about the interaction of PHN and ANT with 2-HP-β/γ-CD, as described in the above experimental results, we ran MD simulations of their complexes too. PHN forms a stable inclusion complex with both hosts; it enters the cavity from the wider side and remains inside until the end of the simulation time (Figure 8 and Figure S3). It is justifiable to mention here that in both cases, PHN is inserted well into the cavity of the hosts. The RMSD values are 1.28 ± 0.20 and 3.23 ± 0.21 (Å) for β and γ, respectively (Table S3), indicating that the PHN complex with the β isomer is more stable than that with the γ isomer. This asserts the significance of the guest molecule’s size and compatibility with the host molecule in the complex formation process.

**Figure 9:**
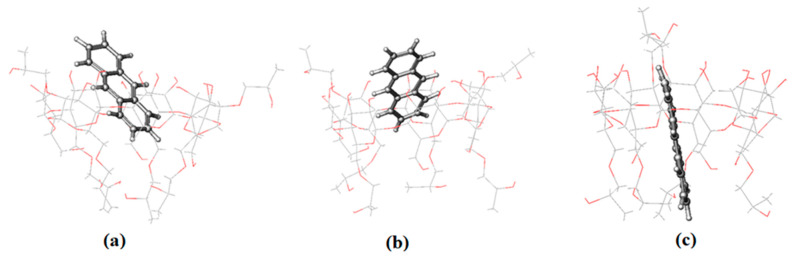
The representative snapshots of the ANT–HP-β-CD obtained during simulation at (**a**) 0 ns, (**b**) 10 ns and (**c**) 30 ns.

The snapshots of ANT-2-HP-β-CD that were taken during simulation reveal that ANT stays inside the host cavity until the end of the simulation time (Figure 9). The docked lowest energy conformer of ANT-2-HP-γ-CD loses its stability immediately after submission; the snapshots taken during the simulation showed that in fewer than 0.2 ns, ANT starts to leave the cavity of the host and moves towards the rim until the end of the simulation time (Figure S4). This happens when the cavity entrance is closed by HP substituents, which move freely. Hence, the docked structure is stereo-structurally unfavorable. In addition to that, the formation of ANT–C–H…O–CD hydrogen bonding with the rim hydroxyl oxygen atom gives rise to this situation. According to the RMSD results (Table S4), the complex of ANT with the β isomer is more stable than that formed with the γ isomer. The cavity size and host–guest complementarity give rise to these results.

From a structural perspective, it is feasible for β-CD to accommodate two ANT molecules simultaneously due to the close proximity of the stacked poly-aromatic cores, with a minimum interlayer distance of approximately 0.35 nm. This distance is significantly smaller than the diameter of the β-CD hydrophobic cavity [44]. However, our simulation did not observe the sharing of the β-CD cavity between two ANT molecules. The hydroxypropyl substituents of the flexible arms caused steric hindrance, which is the main reason for this result.

On the contrary, ANT tends to stay outside the 2-HP-γ-CD cavity. From a structural point of view, ANT is fused linearly, whereas PHN is fused at an angle; this gives PHN five resonance structures. This extra resonance makes the PHN more aromatic and enhances the stability of the π system, being around 6.8 kcal per mole more stable [45]. In this situation, the hydrophobic and hydrogen interaction, size complementarity, and steric hindrance compete with each other to give the most stable trajectory. 

The values of the radius of gyration for all inclusion complexes are compiled in Tables S3 and S4, and they exhibit a further reduction compared to the individual hosts. This observation presents empirical support for the existence of a robust interaction between the two PAHs and the macrocyclic molecules.

**Figure 10:**
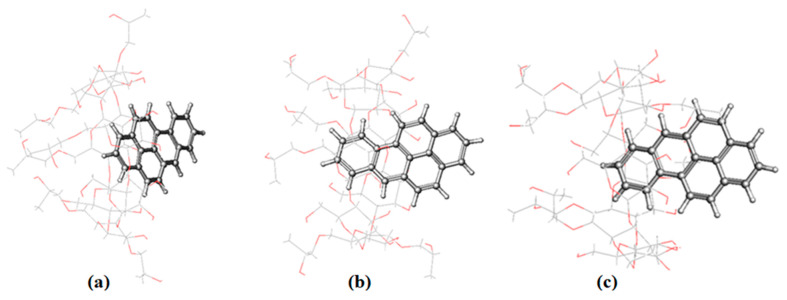
The representative snapshots of BaP-HP-β-CD obtained during simulation at (**a**) 0 ns, (**b**) 15 ns and (**c**) 30 ns.

The submitted structure of the BaP-HP-β/γ-CD complex is not stable. During the simulation time, in less than 1 ns, BaP starts to change its position to adopt the most stable one. The snapshots of the BaP-HP-β/γ-CD complexes collected during the simulation reveal that BaP exits from the nano-cavity of the hosts to enter again via the benzene side and not by the pyrene end, giving a more stable trajectory, as shown in Figure 10 and Figure S5. Although 2-HP-γ-CD has a wider cavity than the 2-HP-β-CD, the RMSD value for the former is larger (Table 4). It has been established that the flexibility of the 8-glucopyranose ring of γ-CD is greater than β-CD, and that the steric hindrance that resulted from freely moving HP arms is the main reason for this result [39]. These flexible arms can rotate freely and sometimes result in the closure of the cavity, consequently prohibiting, to some extent, the complexation process [39]; this partially blocked portal prevents the guests from moving deeper into the cavity [8]. 

The occurrence of two alternative binding mechanisms between the FLT molecule and 2-HP-β/γ-CD can be attributed to the discrepancy in width between the two sides of the FLT molecule [46]. The 2-HP-β-CD cone-shaped cavity forces the FLT to insert itself with its benzene ringside (Figure S6) to maximize the binding constant and minimize the steric hindrance. 2-HP-γ-CD encapsulates the FLT molecules with its narrow side, giving the most stable trajectory (Figure S7). The energy fluctuation associated with the inclusion process suggests that the inclusion complexes assume a geometric arrangement wherein the guest molecule resides within the cavity, thereby enhancing the van der Waals forces.

Table 5 summarizes the radius of gyration, r_gyr_, and the values for the hosts, guests, and their respective inclusion complexes during the simulation. The r_gyr_ values of all complexes exhibit comparability to those of the hosts and typically demonstrate a decrease in magnitude compared to the total of the r_gyr_ values of the individual host and guest. This observation suggests a compact association between the guest and the host.

### 2.4. X-ray Diffraction

**Figure 11:**
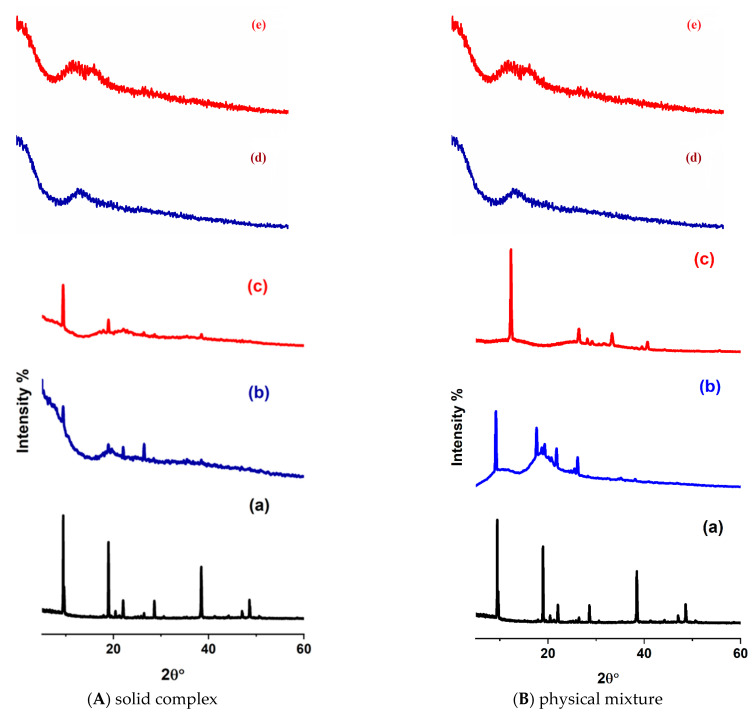
PXRD patterns of (a) PHN with the (b) 2-HP-β-CD and (c) 2-HP-γ-CD solid complex at the left side (**A**), and the physical mixture at the right side (**B**). (d) 2-HP-β-CD and (e) 2-HP-γ-CD.

The PXRD technique was used here to provide evidence for the inclusion of complex formation. Changing the PXRD pattern of the host and guest reflects the change in the phase and the crystallinity of one or both [47,48]. The PXRD patterns of the pure guests, 2-HP-β/γ-CD, and guest–host inclusion complexes were recorded here to investigate the difference in the crystallinity of the structures after the encapsulation process. The PHN PXRD pattern has several high-intensity and sharp peaks at different diffraction angles, at 2θ° = 9.4°, 9.6°, 18.9°, 22.0°, 28.5°, 38.4°, and 48.5°. This proves that this guest is crystalline (Figure 11(Aa)). HP-β/γ-CD is amorphous in its nature. It showed one broad peak in the range of 2θ°~15°–25° (Figure S8 and Figure 11(Ad,Ae)). The formation of the PHN–CDs inclusion complex was confirmed from the diffractogram of the solid complex and physical mixture. Figure 11(Ab,Ac) (left side) reveal that the XRD pattern of the solid inclusion complexes displayed a little broad background under the crystalline peaks. Therefore, adding 2-HP-β/γ-CD to the PHN solution forms new crystalline inclusion complexes with layers resembling some PHN layers.

The diffraction pattern of ANT exhibits characteristic signals at 2θ°~19.4° and less sharp peaks at 9.5°, 29.2°, and 39.2°; this delineates ANT as a crystalline compound (Figure S8a). On the other hand, the solid complex diffractogram of ANT@HP-β/γ-CD shown in Figure S8 (left side) revealed that ANT does not form a solid inclusion complex with 2-HP-γ-CD from their solution (Figure S8c); all 2θ° values coincide with ANT peaks, and they are displayed over the broad host peak (both of them kept their original physical characteristic). Notable changes in the ANT peak intensity were recorded for ANT@HP-β-CD (Figure S8b), implying that the addition of this host encourages the crystals to grow in another direction and has little influence on the stacking behavior of ANT molecules [49]. 

The PXRD for the physical mixtures was recorded to prove that the complexes between the guests and hosts were not formed by just mixing them together (Figure 11B and Figure S8 right sides). One unanticipated finding was that the PXRD patterns of the physical mixtures differed significantly from the solids obtained by the kneading process and even from the pure hosts and guests. 

Previous research has established that PAHs are adsorbed on the surface of natural sediments and soil organic carbon material. This adsorption is similar to the hydrogen bonding interaction and is an exothermic process [50]. It has been noted that PAH adsorbed in the molecules’ hydrophobic domain sites can be entrapped physically in the nano- and microspores through capillary condensation using strong cross-linking bonds [51]. Supramolecular macrocycles have been employed to construct crystalline organic materials (COMs) [52]. COMs are composed of pure organic molecules connected by noncovalent or covalent bonds [53]. Previous studies have confirmed that CDs have intrinsic and extrinsic microporosity [52]. It is well known that CDs have specific adsorption sites (pores) in their solid state. The absorption depends on the size and shape of the adsorption sites and the size and charge of the guests [54]. Hosts interact and stabilize guests by CH·⋯π interaction [55]. Previous studies have documented the phenomenon of guest molecules undergoing diffusion into the lattice voids via a dynamic process [56,57]. Obviously, ANT and PHN could react with CDs in their solid state to form stable crystalline inclusion complexes. Figure 11B and Figure S8, on the right side, demonstrate the diffraction patterns of the physical mixtures of the molecules. PHN and ANT produce a crystalline physical complex with the two hosts, and the new solids have a new crystal structure that differs completely from the pure hosts and guests; hence, a new compound is obtained. 

The physical mixture pattern of PHN@2-HP-β-CD (Figure 11(Bb)) shows a halo PXRD pattern reflecting the amorphous character of the host. Onto this halo structure, sharp peaks were noticed, identical to those of pure PHN, with a recognizable decrease in intensity. In addition, all PHN peaks above 2θ 26° vanished. These missing peaks could originate from stacking guest molecules that lost their stacking after grinding with the host molecules. The PHN@2-HP-γ-CD physical mixture (Figure 11(Bc)) exhibits a new PXRD pattern due to adsorption, which gives new unit cell ordering.

Regarding the physical mixture of ANT@2-HP-β/γ-CD, its diffractograms exhibit new diffraction peaks. The intense sharp peaks originally found at 2θ°~9.5° and 19.4° in the guest sample have disappeared, suggesting the formation of a new crystalline complex [52]. The ANT@2-HP-β-CD physical complex (Figure S8b) is better at ordering than the ANT@2-HP-γ-CD physical mixture (Figure S8c), and the first one exhibits less numbered and higher intense peaks. As solid complexes, the XRD pattern for all molecules under investigation differs from the physical mixture. From these results, we can assume that the interaction occurred due to the extrinsic host porosity.

The diffractograms of BaP@CDs complexes represent amorphous compounds superimposed on a small amount of crystalline material (Figure S9b,c, left side). A new low-intensity peak at 2θ°~33.2° was observed for both complexes, and the BaP low-intensity peaks vanished. It is worth mentioning that a lower peak intensity originates from a small particle size. These results lead to the suggestion of partial inclusion complexation. As the patterns of the two host complexes are almost identical, the host cavity size has no real effect on the solid structure.

A deep examination of the FLT@2-HP-β-CD solid complex patterns (Figure S10b, left side) shows that the position of the peaks coincides with those of pure hosts and guests. No new crystal structure exists, meaning that no inclusion complex formed. A notable decrease in the FLT peak intensity at 2θ°~18.9° was recorded for FLT@2-HP-γ-CD (Figure S10c), which means that the particle size was reduced during complex preparation (Srinivasan and Stalin 2014) [58] A new peak at 2θ°~7.8° was detected, and all minor peaks over 2θ°~25° vanished. These changes in the FLT@2-HP-γ-CD XRD patterns are evidence of new phase formation; consequently, the inclusion complex was formed.

By examining the PXRD pattern of the guests–hosts physical mixture (Figure S9 and Figure 10 right side), we found that the adsorption of the PAHs affects the solid-state architecture of the intermolecular organization [55]. We can see that the PXRD pattern of the BaP@2-HP-β/γ-CD physical mixture differs from that of the guests, hosts, and the solid complex formed by the kneading process (Figure S9 right side). The complex of BaP with β CD has a higher degree of crystallinity and ordering than γCD. On the other hand, an examination of the FLT@2-HP-β/γ-CD diffraction patterns (Figure 10b,c right side) showed that the position of the peaks did not coincide with those of pure hosts and guests; hence, mixing of the solid hosts and guests led to the formation of a new material with the crystalline structure. Both complexes have one highly intense peak at 2θ°~9.5° and 9.3° for β- and γ-CD, respectively. The rest of the minor peaks are broad and have low intensity, indicating the low crystallinity of these compounds. 

It can be concluded that BaP and FLT can form complexes with hydroxypropyl-β/γ-cyclodextrin in solution and in a solid state, but with different crystal structures. The loss of porosity and the collapse of the COMs lattices upon desolvation are the main reasons for the difference between solid and physical mixture complexes [56].

### 2.5. Field Emission Scanning Electron Microscopy (SEM)

**Figure 12:**
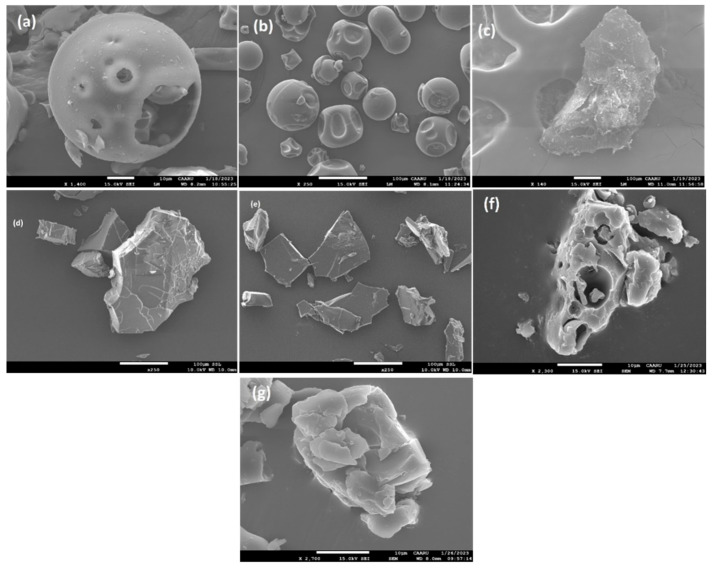
SEM images of (**a**) 2-HP-β-CD, (**b**) 2-HP-γ-CD, (**c**) PHN, (**d**) PHN@2HP-β-CD and (**e**) PHN@2-HPγ-CD solid complexes, and (**f**) PHN@2HP-β-CD and (**g**) PHN@2HP-γ-CD physical mixture.

**Figure 13:**
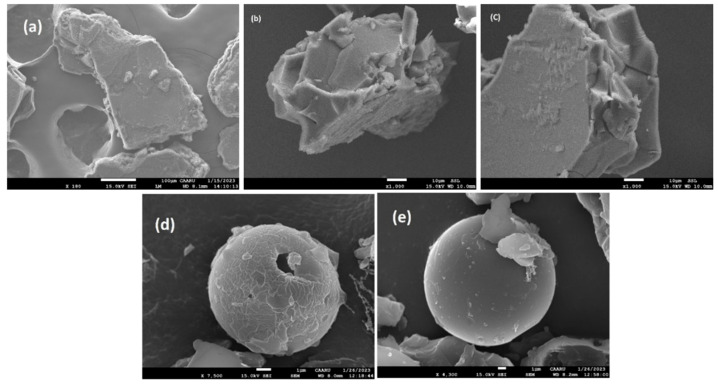
SEM images of (**a**) ANT (**b**), ANT@2-HP-β-CD and (**c**) ANT@2-HP-γ-CD solid complexes, and (**d**) ANT@2-HP-β-CD and (**e**) ANT@2-HP-γ-CD physical mixture.

SEM was employed as a supplementary technique to enhance the analysis of the intricate crystal structures by powder X-ray diffraction (PXRD). SEM was used here to describe the changes in the spatial structure and crystal state [56]. By comparing the differences in the images obtained for the guests, hosts, and the formed complex, we can ascertain the presence of the complex [56]. Transformative shape and particle morphology changes were observed for the 1:1 co-precipitated products of CDs complexes, disclosing the apparent interaction in the solid state (Figure 12 and Figure 13).

The SEM images in Figure 12 show that pure the PHN particles are identified as irregularly formed crystals without sharp edges (Figure 12c). 2-HP-β-CD shows a typical spherical particle structure containing a pore-like cavity (Figure 12a), whereas 2-HP-γ-CD appears as a shrunken spherical particle with a porous appearance (Figure 12b). The crystal structure of PHN@2-HP-β-CD has been exposed to substantial changes. Neither a distinct spherical structure nor a flaky crystal can be observed, but a new irregular shape has emerged, with cracks or rifts on the surface (Figure 12d). PHN@2HP-γ-CD appeared as (sharp edge irregular glass plates) irregular crystals in chaotic assembly (Figure 12c). Another important finding was that the original state of both hosts could not be observed. The physical mixture of PHN@2-HP-β-CD (Figure 12f) has a combination structure resembling the host and guest. In contrast, PHN@2HP-γ-CD (Figure 12g) appears like a clustered husk. As seen from the SEM micrograph (Figure 13b,c), the complexes of ANT@2-HP-β/γ-CD solid complexes appeared disordered solid masses, with no evidence of the presence of the host structure. These structures are apparently similar to the ANT structure (Figure 13a) but are thicker, suggesting that the crystals were growing in one favorable direction in the presence of the hosts. In addition to the changes in the particle shape and morphology observed in the 1:1 solid complexes, the physical mixtures of ANT@2-HP-β/γ-CD (Figure 13d,e) exhibit cyclodextrin shapes with surface variations, indicating that the reaction took place at the surface of the hosts. It is well known that cyclodextrin polymers exhibit the characteristics of porous materials with mesopores and a few nano-cavities [59,60]. These properties contributed to the polymers’ high swelling capacity. Therefore, their networks could be expanded to permit the rapid diffusion of adsorbents during adsorption. Figure S11 shows a representative SEM image for BaP, CDs, and their solid complexes. As shown in the SEM images, BaP appears as an amorphous powder with nano threads (Figure S11c). Meanwhile, pure FLT exists in a tapered woody rod-like structure (Figure S12a).

The SEM micrographs of the complexes of BaP@CDs show nanofibers sprouting out from an amorphous platform (Figure S11d,e). The BaP@2-HP-β-CD physical mixture appeared as cluster masses (Figure S11f), whereas the solid physical mixture of BaP@2-HP-γ-CD (Figure S11g) resembles broken shells or veneers. As we see in the SEM images of the kneaded and physical mixture solid-state systems, the inherent morphology of the basic materials was lost, and it was impossible to distinguish between the individual components. These modifications to the shape and aspect of the particles are evidence of the generation of new solid phases.

The photomicrographs of the FLT@CD samples obtained by SEM are shown in Figure S12. A minor change in the morphology and shape of FLT was observed in the 1:1 co-precipitate products of FLT@2-HP-β-CD (Figure S12b). This observation elucidates a discernible interaction occurring within the solid state. On the other hand, the FLT@2HP-γ-CD solid inclusion complex (Figure S12c) shows neither spherical nor rod-like crystals; instead, the solid inclusion complex exhibits an irregular shape. These morphological changes are clear evidence of the inclusion complex formation. Figure S12d,e represent the FLT@2HP-β/γ-CD complex, and they show changes in the morphology of the CD surface, as most of their pores were filled with the PAH guests.

## 3. Materials and Methods

### 3.1. Materials

All chemicals used in this study were purchased from Sigma Aldrich and were used without further purification. The solvents used to prepare the solutions were of HPLC or spectroscopic grades, and ultrapure water was used for the preparation of the aqueous solutions.

#### Preparation of Solid Inclusion Complexes and Physical Mixtures

##### Co-Precipitation Method

Solid guest–host complexes were prepared using the established standard co-precipitation method [58,60]. The guest and host, at a 1:1 molar ratio, were accurately weighed separately. The saturated guests’ solution was prepared in methanol, and hosts were added to the saturated solution of the guests and sonicated for 20 min. The host solvent was added slowly until the solution became clear. The saturated solution was kept at room temperature for more than 72 h. The obtained residue was collected after the decantation of the other solution. The dried complex was ground to fine powder, and then submitted for analysis. 

##### Preparation of the Physical Mixture

Physical mixtures (PMs) were obtained by pulverizing the 1:1 molar ratio of hosts and guests in a mortar; they were ground for 10 min to ensure a homogeneous blend, and the resultant mixtures were kept in a desiccator for further analysis.

### 3.2. Methods

#### 3.2.1. Fluorescence Measurements

The fluorescence spectra were obtained using a PerkinElmer LS55 fluorescence spectrophotometer (Perkin Elmer, Waltham, MA, USA) equipped with a xenon lamp using a quartz cell with a 1.0 cm path length. All measurements were recorded at room temperature. The stock solutions of the guests were prepared in methanol, ultrapure water was used to prepare the working solutions, and the concentration of this solution was kept at 1.0 μM. In contrast, the concentration of the hosts was 1.0 mM for CDs.

#### 3.2.2. ^1^H NMR Spectroscopy

The nuclear magnetic resonance (NMR) spectroscopy experiments were carried out using a Bruker Avance III HD 700 MHz spectrometer (Bruker, Karlsruhe, Germany) equipped with a 5 mm TCI H/C/N cryoprobe. The proton NMR experiment was run using the zg30 pulse program operating at 700.13 MHz. The acquisition parameters were as follows: a 90° proton pulse width of 8.00 μs, a relaxation delay of 1 s, and 128 scans. The spectrum was recorded at 298 K and processed using TOPSPIN 3.2 software. All host–guest complexes were prepared with deuterated dimethyl sulfoxide (DMSO-d6). All chemical shifts (expressed in ppm scale) are referenced to the solvent signal (DMSO-d6). The ^1^H NMR data were processed with MestReNova v6.0.2-5475 software.

#### 3.2.3. Powder X-ray Diffractometry

Powder X-ray patterns were obtained with Panalytical, X’ Pert PRO X-Ray Diffraction using a copper X-ray source (Kα radiation = 1.5405 Å), a voltage of 45 kV and a 40 mA current, with a scan speed of 2θ = min^−1^ and an Xcelerator X-ray detector. The powdered samples were analyzed over an angular range of 5° to 70° 2θ°. The diffraction patterns were acquired using X-pert Data Collector software and processed using Origin 2022 software.

#### 3.2.4. Field Emission Scanning Electron Microscopy SEM

The surface characteristics of the pure guests, hosts, and prepared complex were analyzed using Field Emission Scanning Electron Microscopy (SEM). The SEM analysis was performed using JSM-7600F Schottky (Jeol Company, Tokyo, Japan). The SEI resolution was 1.0 nm (15 kv) to 1.5 nm (Ikv), the accelerating voltage was 0.1 kV to 30 kV, and the probe current was 1 pA to 200 nA. Magnification 25 to 1,000,000 (on an image size of 120–90 mm). The sample was placed, without further treatment, on a sample rack after coating it with platinum to improve visibility.

#### 3.2.5. Molecular Modeling

The initial geometry of PHN and ANT was optimized using the DFT-B3LYP method using a 6-31G* basis set. On the other hand, the structures of the hosts were extracted from the crystallographic parameters provided by the Structural Data Base System of the Cambridge crystallographic data center and were optimized by minimizing their energy using the PM6 semi-empirical method and MOPAC 2012 [61]. 

Molecular docking experiments were performed using the Autodock program (version 4.2) [43]. In this work, we used a Lamarckian genetic algorithm (LGA) for docking the guest into the host’s cavity to generate the inclusion complexes of the most appropriate conformation. Generally, Autodock defines the conformational space by implementing grids over all the possible search spaces. The initial torsions and positions of the guests were generated randomly, and Autodock tools were utilized to extract the optimum conformers using cluster analysis for all inclusion complexes, using a cutoff of 1.0 Å root mean square deviation (RMSD). The lowest energy structures were further optimized using the PM6 semi-empirical model.

The molecular dynamic (MD) simulations were carried out using the Desmond molecular simulations package, as distributed by the Schrodinger 2015 suite of programs. The OPLS_2005 all-atom force field with explicit solvent (TIP3P water model) was used throughout the calculations. Simulations were run with periodic boundary conditions in an orthorhombic box with the solute viz., placed in the middle at a 20 Å distance from each of the box’s edges. The SHAKE algorithm constrains covalent bonds between hydrogen and heavy atoms. The Ewald smooth particle mesh (PME) method dealt with long-range electrostatic interactions [62]. The solvated molecules were subjected to sequential restraint solvent–solute minimizations and short MD simulations on the NVT-NPT ensembles (as implemented in the default relaxation protocol in Desmond) coupled to the Berendsen thermostat. Finally, the production run was NPT run at 300 K and 1 bar. The simulations were then analyzed by the “simulation event analysis” module in the Schrodinger 2015 suite.

## 4. Conclusions

In this study, we examined the potential of 2-HP-β/γ-CD to form stable inclusion/association complexes with ANT, PHN, BaP, and FLT, both in solution and the solid state. The significant increase in the fluorescence emission of these aromatic compounds provides evidence for the thermodynamically favorable transition of the non-polar guest from a polar aqueous medium to a non-polar environment within the cavity or, in certain instances, adjacent to the external walls of the guest. This transition is expedited by non-covalent bonding interactions, which serve to stabilize the guest–host complex. The results consistently demonstrate the formation of host–guest complexes in a 1:1 ratio. The analysis of the ^1^H NMR data reveals that the protons of the guest molecule experience distinct electronic and magnetic environments, as evidenced by observable changes in the chemical shifts. Additionally, molecular dynamics simulations confirm the stability of all complexes in aqueous media throughout the simulation period. PHN enters the nanocavity of the hosts, whereas the hydroxypropyl arms of the γ-cyclodextrin block the ANT molecule from entering the cavity. The inclusion complex between HP-β-CD and FLT is more stable than that between HP-β-CD and BaP. The solid complexes were produced using the co-precipitation process and subsequently analyzed using PXRD and SEM techniques. These solid complexes were compared with the solid physical mixtures. The results obtained indicate that stable complexes were obtained in both cases. The observation of distinct diffraction patterns, which differ from those observed in the pure host and guest, provides compelling evidence of the creation of an inclusion complex. All guests form different morphological complexes in the presence of the hosts. We concluded that host–guest interactions affect the morphology, photoluminescence, and magnetic properties of the guests. The supramolecular organization of these molecules in the solid state can have a crucial influence on their capacity for charge transfer. These findings expand our understanding of the supramolecular chemistry of these species, opening the door for the further development of novel PAH quantification protocols.

## Figures and Tables

**Table 1:** Parameters of the fit of the Bensi–Hildebrand equation to the fluorescence of PHN, ANT, BaP and FLT in the presence of 2-HP-β/γ-CD.

PHN:[Host] Complex	Molar Ratio	Binding Constant M^−1^	Equation	R^2^
2-HP-β-CD	1:1	85 ± 12	Y = (5.5 × 10^−5^)X + 0.0046	0.9973
2-HP-γ-CD	1:1	49 ± 29	Y = (1.02 × 10^−4^)X + 0.005	0.984
ANT:[Host] complex				
2-HP-β-CD	1:1	502 ± 46	Y = (1.00 × 10^−4)^X + 0.0147	0.9746
2-HP-γ-CD	1:1	289 ±44	Y = (4.00 × 10^−7^)X + 0.0949	0.9994
BaP:[Host] complex				
2-HP-β-CD	1:1	1500 ± 200	Y = (4.0 × 10^−7^)X + 0.0006	0.9994
2-HP-γ-CD	1:1	94 ± 28	Y = (3.67 × 10^−6^)X + 0.0003	0.995
FLT:[Host] complex				
2-HP-β-CD	1:1	1060 ± 60	Y = (9.06 × 10^−6^)X + 0.0095	0.9867
2-HP-γ-CD	1:1	0.14 ± 14	Y = (8.7 × 10^−5^)X + 1.1 × 10−5	0.992

**Table 2:** The ^1^H-NMR shifts between free and complexed protons in the BaP-HP-β/γ-CD complex.

Proton	δ_(Free)_/ppm	δ_(B[a]P-HP-β-CD)_/ppm	Δδ_(δComplex − δFree)_/ppm	δ_(B[a]P-HP-γ-CD)_/ppm	Δδ_(δComplex − δFree)_/ppm
H1′	8.4040	8.3960	−0.0080	8.3980	−0.0060
H2′	8.2280	8.2180	−0.0100	8.2200	−0.0080
H3′	8.3760	8.3660	−0.0100	8.3680	−0.0080
H4′	8.0700	8.0580	−0.0120	8.0610	−0.0090
H5′	8.1320	8.1240	−0.0080	8.1260	−0.0060
H6′	8.7170	8.7100	−0.0070	8.7130	−0.0040
H7′	8.4600	8.4530	−0.0073	8.4555	−0.0045
H8′	7.8606	7.8500	−0.0105	7.8520	−0.0085
H9′	7.9025	7.8930	−0.0095	7.8950	−0.0075
H10′	9.2525	9.2410	−0.0115	9.2450	−0.0075
H11′	9.2275	8.2170	−0.0105	9.2200	−0.0075
H12′	8.4725	8.4660	−0.0065	8.4685	−0.0040

**Table 3:** The ^1^H-NMR shifts between free and complexed protons in FLT- HP-β/γ-CD complex.

Proton	δ_(free)_/ppm	δ_(FLT-HP-β-CD)_/ppm	Δδ_(δComplex − δfree)_/ppm	δ_(FLT-HP-γ-CD)_/ppm	Δδ_(δComplex − δfree)_/ppm
H1’	8.1325	8.1350	0.0025	8.1360	0.0035
H2’	7.715	7.7170	0.0020	7.7180	0.0030
H3’	7.9620	7.9630	0.0010	7.9640	0.0020
H4’	8.057	8.0580	0.0010	8.0590	0.0020
H5’	7.4275	7.4295	0.0020	7.4295	0.0020

**Table 4:** Average value of RMSD and radius of gyration obtained from molecular dynamics trajectories for various compounds used in the study.

Compound	RMSD (Å)	r_gyr_ (Å)
BaP-2-HP-β-CD	2.57 ± 0.33	6.35 ± 0.06
BaP	0.20 ± 0.04	3.18 ± 0.01
2-HP-β-CD	1.86 ± 0.21	6.58 ± 0.11
BaP-2-HP-γ-CD	3.32 ± 0.48	6.57 ± 0.12
BaP	0.18 ± 0.04	3.18 ± 0.01
2-HP-γ-CD	3.07 ± 0.37	6.76 ± 0.17

**Table 5:** Average value of RMSD and radius of gyration obtained from molecular dynamics trajectories for various species.

Compound	RMSD (Å)	r_gyr_ (Å)
FLT-2-HP-β-CD	1.67 ± 0.21	6.35 ± 0.07
FLT	0.19 ± 0.04	2.74 ± 0.01
2-HP-β-CD	1.57 ± 0.19	6.66 ± 0.09
FLT-2-HP-γ-CD	3.00 ± 0.37	6.57 ± 0.09
FLT	0.18 ± 0.03	2.74 ± 0.01
2-HP-γ-CD	3.12 ± 0.36	6.85 ± 0.11

## Data Availability

The original data presented in the study are openly available in the Supporting Information of this manuscript.

## Supplementary Materials

The supporting information can be downloaded at: https://www.mdpi.com/article/10.3390/molecules29112535/s1, Figure S1: Fluorescence spectra of (0) FLT, 1.0 × 10^−6^ M with increasing concentration of (a) 2-HP-β-CD 1.0 × 10^−4^ M → 5.5 × 10^−4^ M. (b) 2-HP-γ-CD 1.0 × 10^−3^ M → 6.0 × 10^−3^ M; Figure S2: A partial 1H-NMR spectra for (B)FLT-HP-β-CD, (C) FLT-HP-γ-CD complex compared to (a) free FLT in DMSO. The molar ratio of HP-β/γ-CD: guest is 1:1; Figure S3: The representative snapshots of PHN-HP-γ-CD obtained during simulation at (a) 0 ns (b) 15 ns (c) 30 ns; Figure S4: The representative snapshots of ANT-HP-γ-CD obtained during simulation at (a) 0 ns (b) 30 ns; Figure S5: The representative snapshots of BaP@2-HP-γ-CD obtained during simulation at (a) 0 ns (b) 4 ns (c) 8 ns (d) 30 ns; Figure S6: The representative snapshots of FLT- HP-β-CD obtained during simulation at (a) 0 ns (b) 5 ns (c) 15 ns (d) 30 ns; Figure S7: The representative snapshots of FLT@2-HP-γ-CD obtained during simulation at (a) 0 ns (b) 3 ns (c) 5 ns (d) 30 ns; Figure S8: PXRD patterns of (a) ANT physical mixture with (b) 2-HP-β-CD (c) 2-HP-γ-CD at left side and physical mixture at right side; Figure S9: PXRD patterns of (a) BaP with (b) 2-HP-β-CD (c) 2-HP-γ-CD physical mixture at right side and solid complex at left side; Figure S10: PXRD patterns of (a) FLT with (b) 2-HP-β-CD (c) 2-HP-γ-CD solid complex at right side physical mixture at left side and; Figure S11: SEM images of (a) 2-HP-β-CD (b) 2-HP-γ-CD (c) BaP (d) BaP@2-HP-β-CD (e) BaP@2-HPγ-CD Solid complexes and (f) BaP@2-HP-β-CD (g) BaP@2-HPγ-CD physical mixture; Figure S12: SEM images of (a) FLT (b) FLT@2-HP-β-CD (c) FLT@2HP-γ-CD Solid complexes and (b) FLT@2-HP-β-CD (c) FLT@2HP-γ-CD physical mixture; Table S1: The 1H-NMR shifts between free and complexed protons in PHN- HP-β/γ-CD complex; Table S2: The 1H-NMR shifts between free and complexed protons in ANT- HP-β/γ-CD complex.
