# Supplementary material for: Polyaromatic Hydrocarbon Inclusion Complexes with 2-Hydroxylpropyl-β/γ-Cyclodextrin: Molecular Dynamic Simulation and Spectroscopic Studies
Source: Molecules. 2024 May 28;29(11):2535. doi: 10.3390/molecules29112535 (PMC11173409; doi:10.3390/molecules29112535)
Supplement: Supplementary file 1 [file molecules-29-02535-s001.zip › molecules-3019773-supplementary.pdf]

Polyaromatic hydrocarbon inclusion complexes with 2-hydroxylpropyl-β/γ-cyclodextrin: molecular dynamic simulation and spectroscopic studies.

Norah S. Alsadun[1*], Amira A. Alfadil[2,3,4], Abdalla A. Elbashir[1,3*] FakhrEldin O. Suliman[2], Mei Musa Ali Omar[5], Amel Y. Ahmed[1]

[1] Department of Chemistry, College of Science, King Faisal University, Al-Hofuf, Al-Ahsa, 31982 Saudi Arabia

[2] Department of Chemistry, College of Science, Sultan Qaboos University, Box 36, Al-Khoud 123, Oman.

[3] University of Khartoum, Faculty of Science, Department of Chemistry, Khartoum, 11114 Sudan.

[4] Sudan University of Science and Technology, College of Science, Department of Scientific Laboratories, Khartoum, Sudan.

[5] Department of Chemistry, Central Laboratory, Ministry of Higher Education & Scientific Research, P. O. Box Office 7099, Khartoum, Sudan

*Correspondence to: Dr. Norah Sadun Alsadun, e-mail: nalsadoun@kfu.edu.sa
Professor Abdalla A. Elbashir, e-mail: aaeahmed@kuf.edu.sa

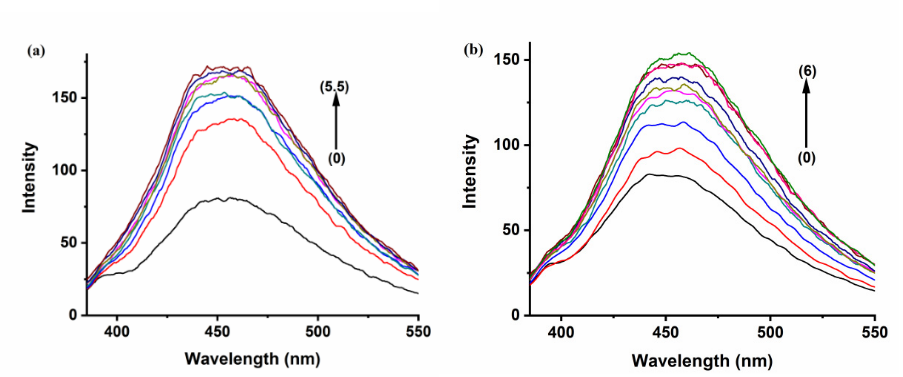

Fig. S1. Fluorescence spectra of (0) FLT, 1.0×10^{-6} M with increasing concentration of (a) 2-HP-β-CD 1.0×10^{-4} M → 5.5×10^{-4} M. (b) 2-HP-γ-CD 1.0×10^{-3}M →6.0×10^{-3}M.

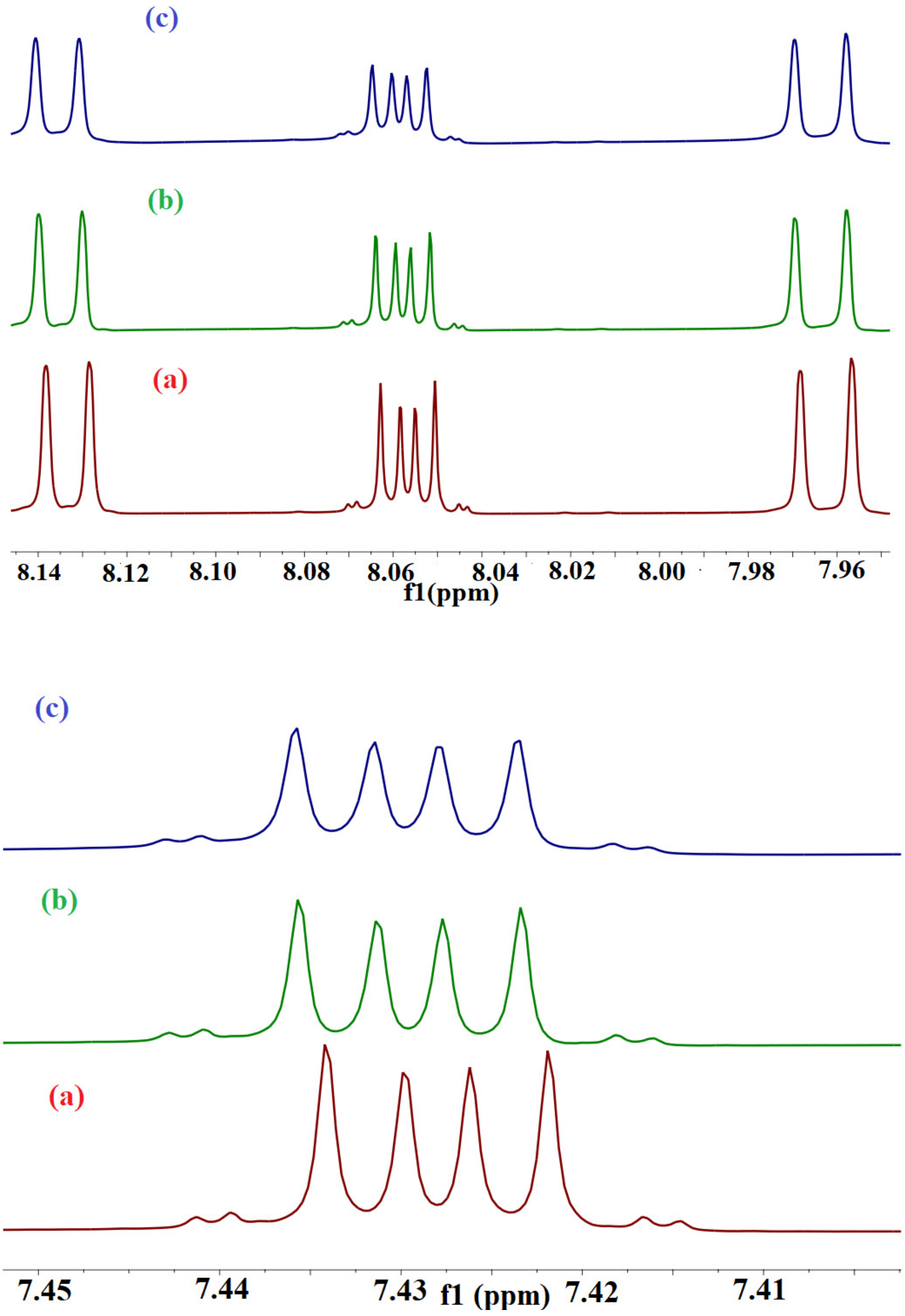

Fig. S2. A partial ^{1}H-NMR spectra for (B)FLT-HP-β-CD, (C) FLT-HP-γ-CD complex compared to (a)free FLT in DMSO. The molar ratio of HP-β/γ-CD: guest is 1:1.

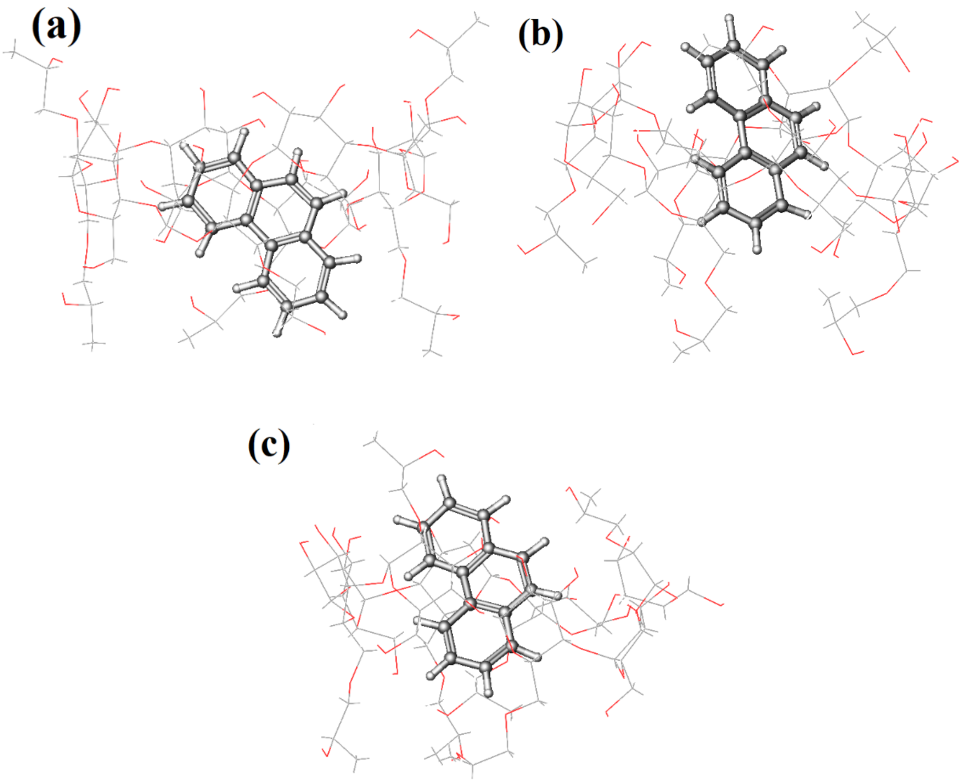

Fig. S3. The representative snapshots of PHN-HP-γ-CD obtained during simulation at (a) 0 ns (b) 15 ns (c) 30 ns

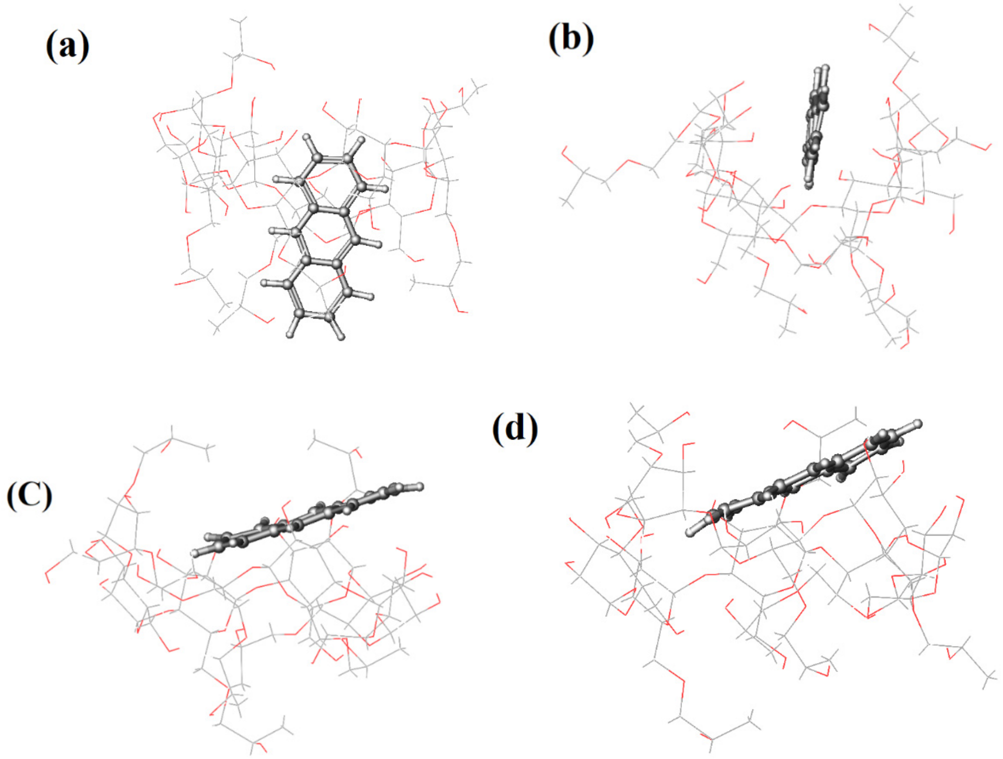

Fig. S4. The representative snapshots of ANT-HP-γ-CD obtained during simulation at (a) 0 ns (b) 30 ns.

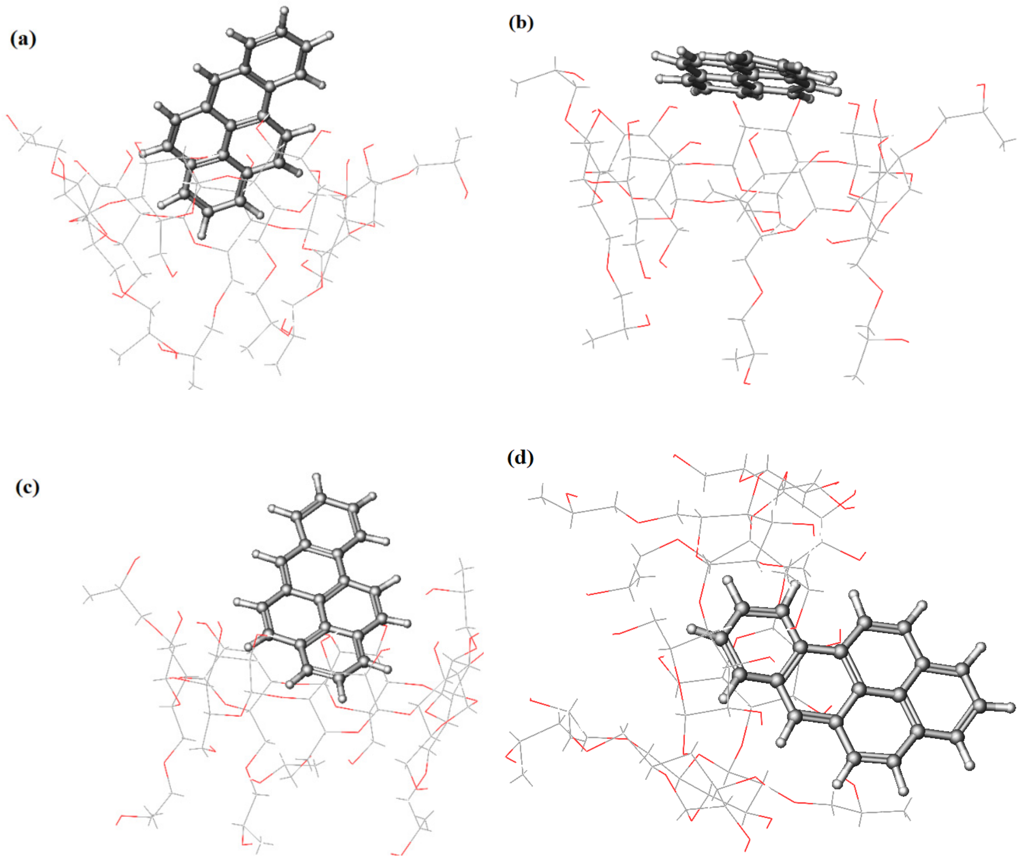

Fig. S5. The representative snapshots of BaP@2-HP-γ-CD obtained during simulation at (a) 0 ns (b) 4 ns (c) 8 ns (d) 30 ns.

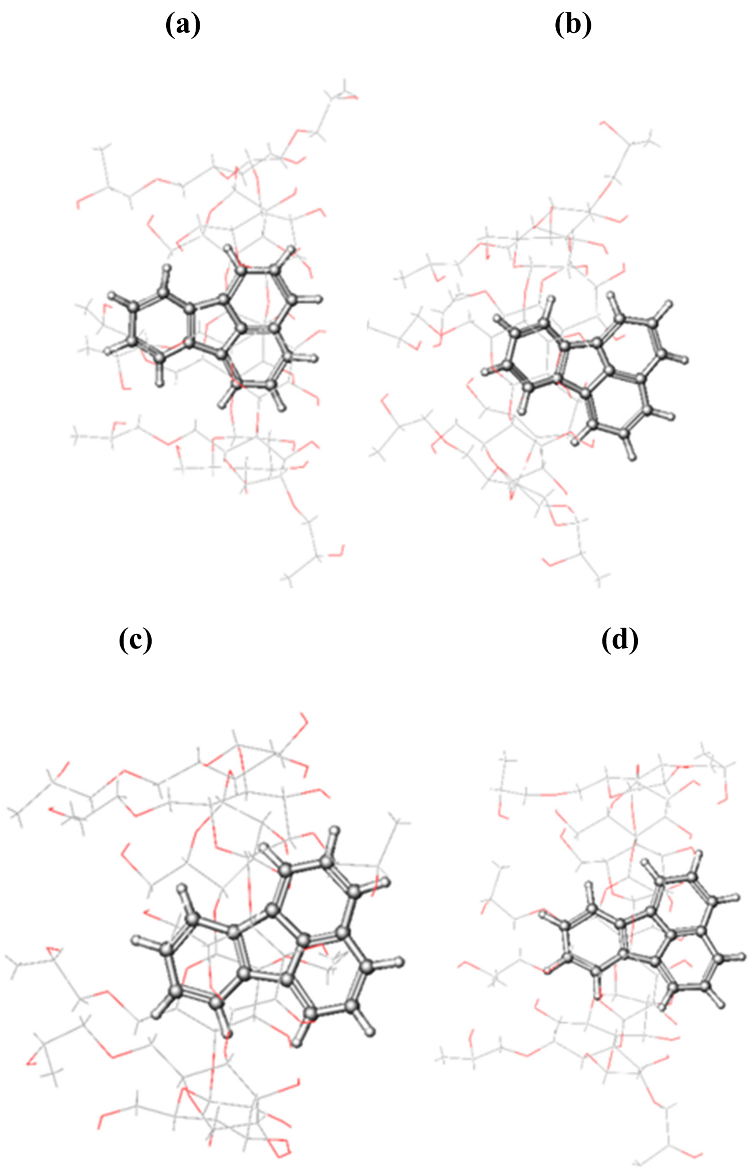

Fig. S6. The representative snapshots of FLT- HP-β-CD obtained during simulation at (a) 0 ns (b) 5 ns (c) 15 ns (d) 30 ns.

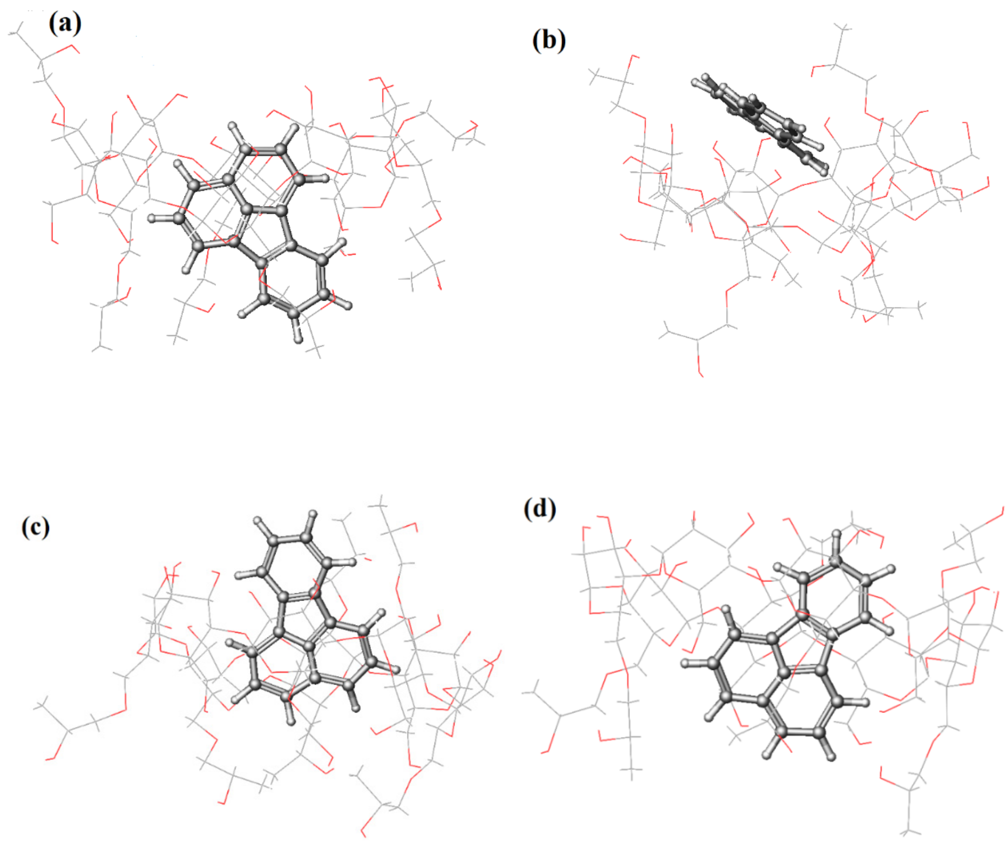

Fig. S7. The representative snapshots of FLT@2-HP-γ-CD obtained during simulation at (a) 0 ns (b) 3 ns (c) 5 ns (d) 30 ns.

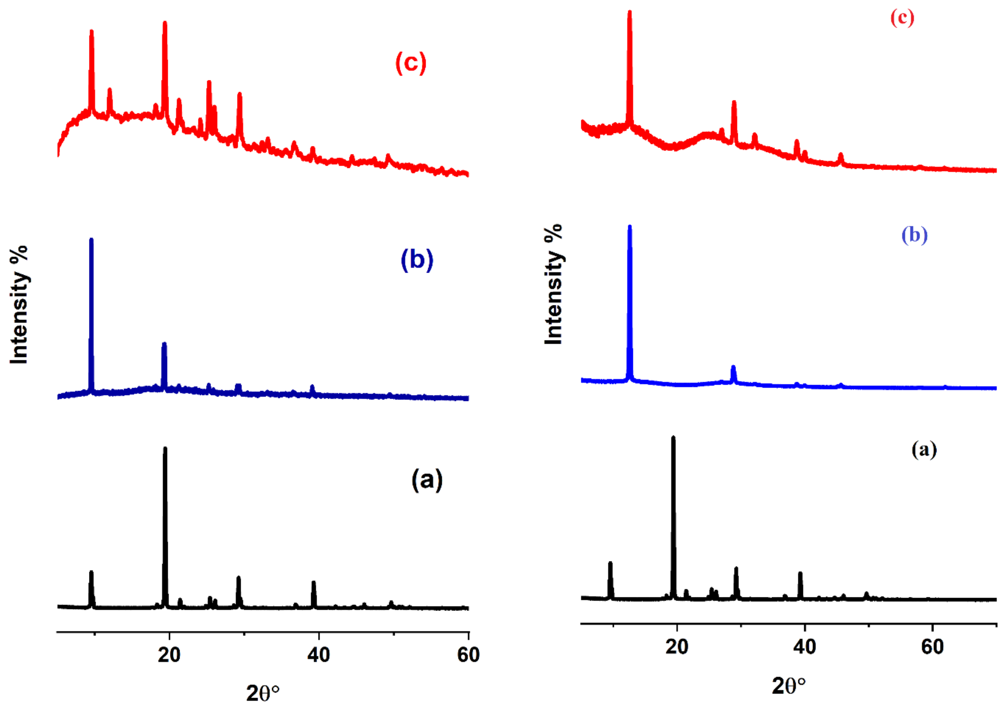

Fig. S8. PXRD patterns of (a) ANT physical mixture with (b) 2-HP-β-CD (c) 2-HP-γ-CD at left side and physical mixture at right side.

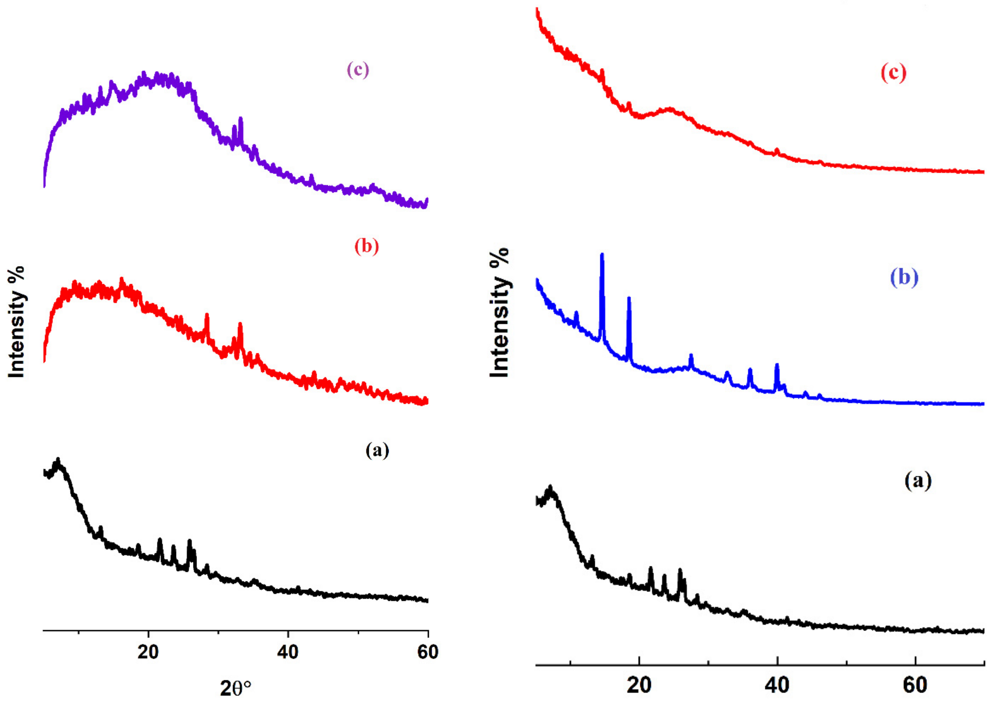

Fig. S9. PXRD patterns of (a) BaP with (b) 2-HP-β-CD (c) 2-HP-γ-CD physical mixture at right side and solid complex at left side.

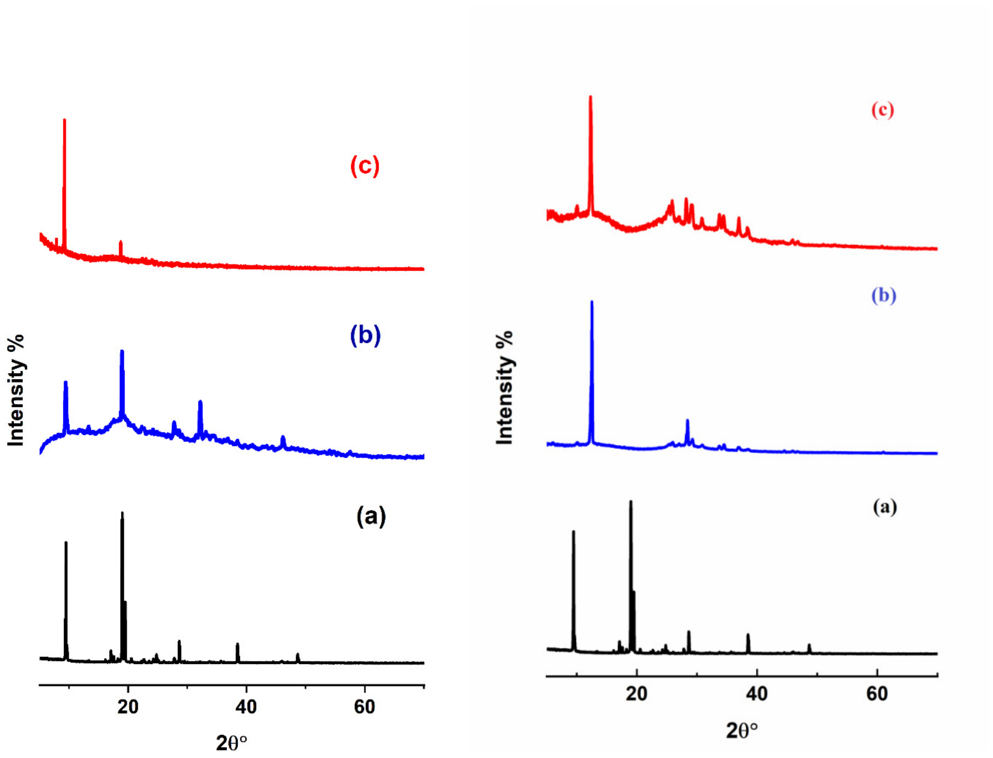

Fig. S10. PXRD patterns of (a) FLT with (b) 2-HP-β-CD (c) 2-HP-γ-CD solid complex at right side physical mixture at left side and.

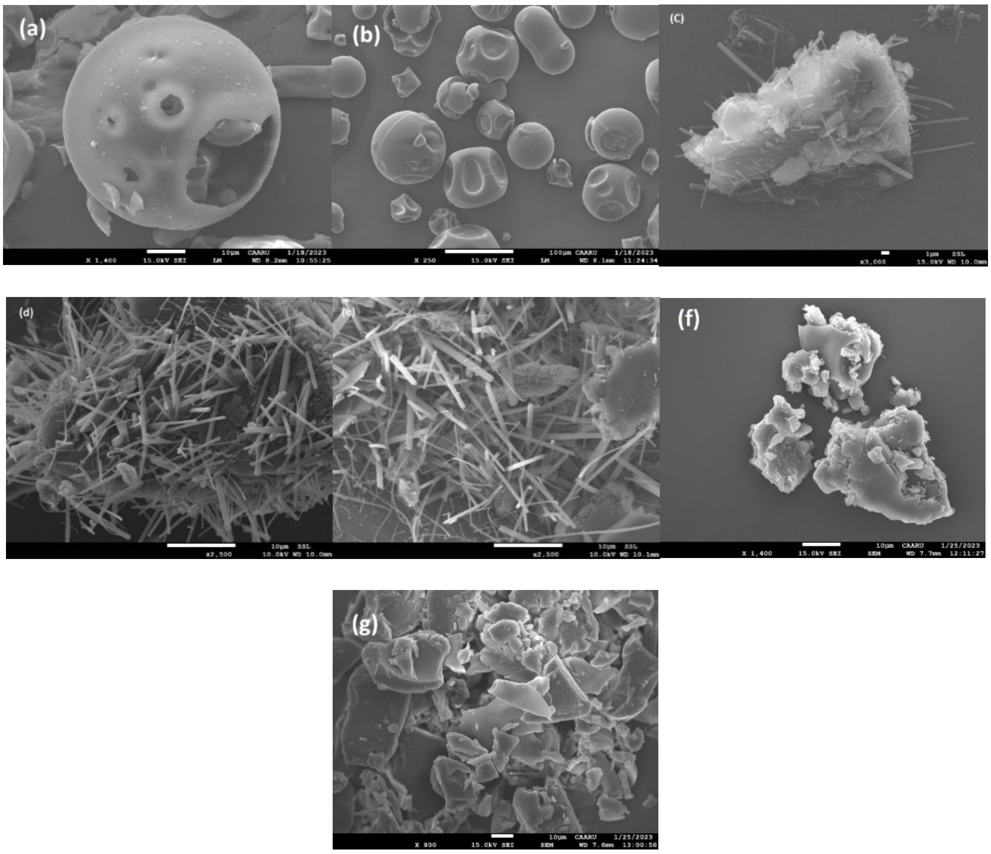

Fig. S11. SEM images of (a) 2-HP-β-CD (b) 2-HP-γ-CD (c) BaP (d) BaP@2-HP-β-CD (e) BaP@2-HPγ-CD Solid complexes and (f) BaP@2-HP-β-CD (g) BaP@2-HPγ-CD physical mixture.

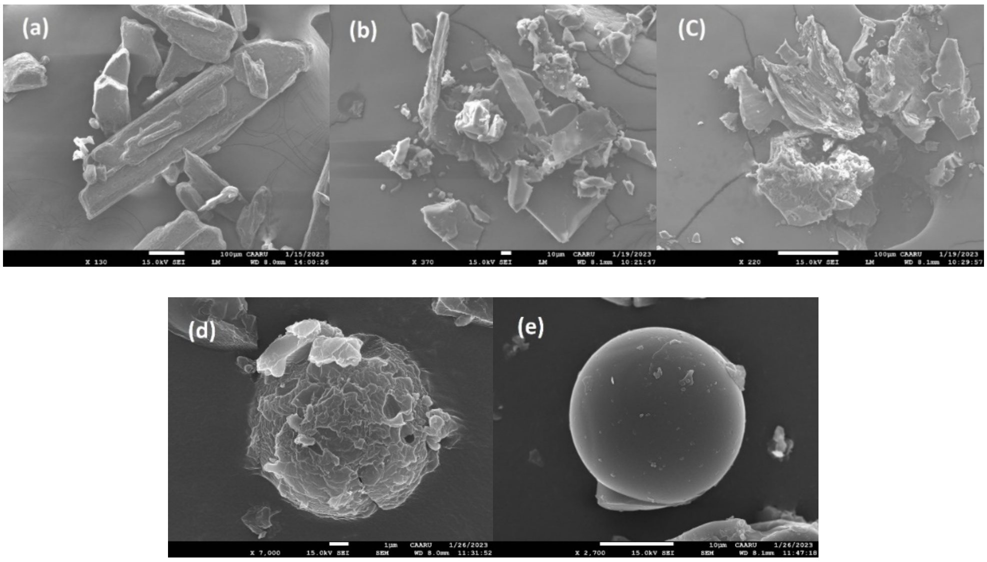

Fig. S12. SEM images of (a) FLT (b) FLT@2-HP-β-CD (c) FLT@2HP-γ-CD Solid complexes and (b) FLT@2-HP-β-CD (c) FLT@2HP-γ-CD physical mixture.

Table S1. The ^{1}H-NMR shifts between free and complexed protons in PHN- HP-β/γ-CD complex.					
**Proton**	**$\delta_{(free)}$ /ppm**	**$\delta_{(PHN-HP-\beta-CD)}$ /ppm**	**$\Delta\delta_{(\delta Complex-\delta free)}$ /ppm**	**$\delta_{(PHN-HP-\gamma-CD)}$ /ppm**	**$\Delta\delta_{(\delta Complex-\delta free)}$ /ppm**
**H1′**	8.8250	8.8310	0.0060	8.8300	0.0050
**H2′**	7.6985	7.7015	0.0030	7.7015	0.0030
**H3′**	7.6500	7.6525	0.0025	7.6530	0.0030
**H4′**	7.9880	7.9910	0.0030	7.9900	0.0100
**H5′**	7.8445	7.8475	0.0030	7.8470	0.0025

Table S2. The ^{1}H-NMR shifts between free and complexed protons in ANT- HP-β/γ-CD complex.					
**Proton**	**$\delta_{(free)}$/ppm**	**$\delta_{(ANT-HP-\beta-CD)}$/ppm**	**$\Delta\delta(\delta_{Complex}-\delta_{free})$/ppm**	**$\delta_{(ANT- HP-\gamma-CD)}$/ppm**	**$\Delta\delta_{(\delta Complex-\delta free)}$/ppm**
**H1'**	8.5750	8.5755	0.0005	8.5760	0.0010
**H2'**	8.0865	8.0870	0.0005	8.0880	0.0015
**H3'**	7.5150	7.5155	0.0005	7.5160	0.0010

**Table S3.** The average value of RMSD and radius of gyration obtained from molecular dynamics trajectories for various species.		
**Compound**	**RMSD (Å)**	**r_{gyr} (Å)**
**PHN-HP-β-CD**	1.28 ± 0.20	6.31 ± 0.07
PHN	0.21 ± 0.04	2.62 ± 0.01
HP-β-CD	1.55 ± 0.17	6.59 ± 0.08
**PHN-HP-γ-CD**	3.23 ± 0.21	6.49 ± 0.10
PHN	0.18 ± 0.04	2.62 ± 0.01
HP-γ-CD	3.60 ± 0.22	6.75 ± 0.11

**Table S4. Average value of RMSD and radius of gyration obtained from molecular dynamics trajectories for various species.**		
**Compound**	**RMSD (Å)**	**r_{gyr} (Å)**
**ANT-HP-β-CD**	1.67 ± 0.32	6.33 ± 0.07
**ANT**	0.20 ± 0.04	2.75 ± 0.01
**HP-β-CD**	1.68 ± 0.23	6.59 ± 0.09
**ANT-HP-γ-CD**	3.28 ± 0.38	6.50 ± 0.10
**ANT**	0.16 ± 0.03	2.75 ± 0.01
**HP-γ-CD**	3.11 ± 0.41	6.69 ± 0.11
